# From monkeys to humans: observation-based EMG brain–computer interface decoders for humans with paralysis

**DOI:** 10.1088/1741-2552/ad038e

**Published:** 2023-11-01

**Authors:** Fabio Rizzoglio, Ege Altan, Xuan Ma, Kevin L Bodkin, Brian M Dekleva, Sara A Solla, Ann Kennedy, Lee E Miller

**Affiliations:** 1 Department of Neuroscience, Northwestern University, Chicago, IL, United States of America; 2 Department of Biomedical Engineering, Northwestern University, Evanston, IL, United States of America; 3 Rehab Neural Engineering Labs, Department of Physical Medicine and Rehabilitation, University of Pittsburgh, Pittsburgh, PA, United States of America; 4 Department of Physics and Astronomy, Northwestern University, Evanston, IL, United States of America; 5 Shirley Ryan AbilityLab, Chicago, IL, United States of America; 6 Department of Physical Medicine and Rehabilitation, Northwestern University, Chicago, IL, United States of America

**Keywords:** brain computer interface, monkey, paralyzed human, EMG decoding, transfer learning

## Abstract

*Objective*. Intracortical brain–computer interfaces (iBCIs) aim to enable individuals with paralysis to control the movement of virtual limbs and robotic arms. Because patients’ paralysis prevents training a direct neural activity to limb movement decoder, most iBCIs rely on ‘observation-based’ decoding in which the patient watches a moving cursor while mentally envisioning making the movement. However, this reliance on observed target motion for decoder development precludes its application to the prediction of unobservable motor output like muscle activity. Here, we ask whether recordings of muscle activity from a surrogate individual performing the same movement as the iBCI patient can be used as target for an iBCI decoder. *Approach*. We test two possible approaches, each using data from a human iBCI user and a monkey, both performing similar motor actions. In one approach, we trained a decoder to predict the electromyographic (EMG) activity of a monkey from neural signals recorded from a human. We then contrast this to a second approach, based on the hypothesis that the low-dimensional ‘latent’ neural representations of motor behavior, known to be preserved across time for a given behavior, might also be preserved across individuals. We ‘transferred’ an EMG decoder trained solely on monkey data to the human iBCI user after using Canonical Correlation Analysis to align the human latent signals to those of the monkey. *Main results*. We found that both direct and transfer decoding approaches allowed accurate EMG predictions between two monkeys and from a monkey to a human. *Significance*. Our findings suggest that these latent representations of behavior are consistent across animals and even primate species. These methods are an important initial step in the development of iBCI decoders that generate EMG predictions that could serve as signals for a biomimetic decoder controlling motion and impedance of a prosthetic arm, or even muscle force directly through functional electrical stimulation.

## 1. Introduction

Intracortical brain–computer interfaces (iBCIs) promise to restore voluntary movement to persons with paralyzed limbs. A kinematic iBCI uses a ‘decoder’ to transform neural activity into signals that can be used to control a cursor or a robotic limb (Serruya *et al* 2002, Taylor *et al* 2002a, Carmena *et al* 2003, Musallam *et al* 2004, Collinger *et al* 2013, Wodlinger *et al* 2014). Conversely, a decoder in a biomimetic iBCI aims to reproduce the original pattern of muscle activation that neural activity would have driven. In a proof of principle, electromyographic (EMG) predictions were used to reanimate the temporarily paralyzed muscles of monkeys’ hands by using them to control the intensity of functional electrical stimulation (FES) (Ethier *et al* 2012). FES is used in a variety of clinical applications for both lower (Kralj *et al* 1988, Triolo *et al* 1996, Popovic *et al* 2001, Shiyu *et al* 2020) and upper extremities (Eraifej *et al* 2017, Marquez-Chin and Popovic 2020, Taylor *et al* 2002b). None of the current clinical applications are brain-controlled, but two recent studies implemented rudimentary brain-controlled FES of hand (Bouton *et al* 2016) or arm and hand (Ajiboye *et al* 2017) indirectly, driven by a simple grasp classifier or a kinematic decoder respectively.

Decoders are typically trained with supervised learning methods, tuning parameters to minimize the error between a decoder-generated transformation of neural activity and an observed motor-related output (Hochberg *et al* 2006, Collinger *et al* 2013, Willett *et al* 2021). For iBCI applications developed in intact monkeys, decoders can be trained directly on recorded M1 firing rates and movement (or muscle activity). However, for application to a paralyzed human with no motor output, this is not possible. In this case, the standard approach is to use an ‘observation-based’ strategy, in which a subject observes a cursor or a target performing a movement. Observation-based decoders are then trained to map M1 activity into the observed (and presumably attempted) kinematic trajectory (Hochberg *et al* 2006, Willett *et al* 2019). But this approach is not feasible for a decoder that maps neural activity into EMGs, which cannot be observed.

There are two potential solutions to this problem, both of which involve a ‘target’ human iBCI user with a paralyzed arm attempting the same type of movement performed by another ‘source’ subject, human or even monkey. One approach is to create a direct mapping between the iBCI user’s recorded neural signals and the recorded ‘source’ EMG signals. This approach is similar to an observation-based decoder, except the neural signals are mapped to the recorded EMG signals instead of observed endpoint kinematics. An advantage of this ‘direct decoding’ approach is its simplicity. It essentially mimics the well-established observation approach for movement-related EMG signals that cannot actually be observed. A disadvantage is that it necessarily requires a close match between the movements (or motor actions, more generally) attempted by the source and target individuals.

An alternative solution might be to use a single, fixed decoder trained on a source monkey, where both neural and EMG activity can be recorded, and then inputting to that decoder the M1 signals recorded from the target iBCI user. This would obviously not be possible using single-neuron firing rates as inputs, since individual neurons differ completely for the source and target subjects. A possible solution might be found through the use of the latent signals that exist within a low-dimensional ‘neural manifold’ computed from neural firing rates (Churchland *et al* 2012, Cunningham and Yu 2014, Gao and Ganguli 2015, Gallego *et al* 2017). Several groups have shown that, despite being embedded in different neural spaces, the latent signals computed from neural recordings made in different experimental sessions can be ‘aligned’ to one another. By this method, a fixed decoder using aligned latent signals as inputs can remain stable, despite changes in the recorded neurons, for many months (Farshchian *et al* 2019, Degenhart *et al* 2020, Gallego *et al* 2020, Karpowicz *et al* 2022, Ma *et al* 2023). Similar alignment methods might allow the latent signals from two monkeys or from a human and a monkey to be aligned to each other, enabling what we call ‘transfer decoding’.

We tested these two approaches using data collected from three monkeys performing a version of the standard center-out task requiring production of forces in eight directions about the wrist. After developing and testing the alignment methods across monkeys, we applied them to a source monkey and a target human with a paralyzed arm. Both direct and transfer decoding produced accurate EMG predictions, though the direct method was consistently higher. Critically, the accuracy of EMG predictions was tied closely to the similarity between latent neural signals of the source and target. We consider these findings to be supportive of future clinical implementations enabling the use of more biomimetic iBCI decoders that could either directly restore voluntary arm movements through FES or allow the control of an anthropomorphic prosthetic arm’s motion and impedance.

## 2. Methods

### 2.1. Monkey task and recordings

Neural and muscle activity data were collected from three adult male rhesus macaque monkeys. All surgical and experimental procedures were approved by the Institutional Animal Care and Use Committee of Northwestern University.

**Figure 1.**
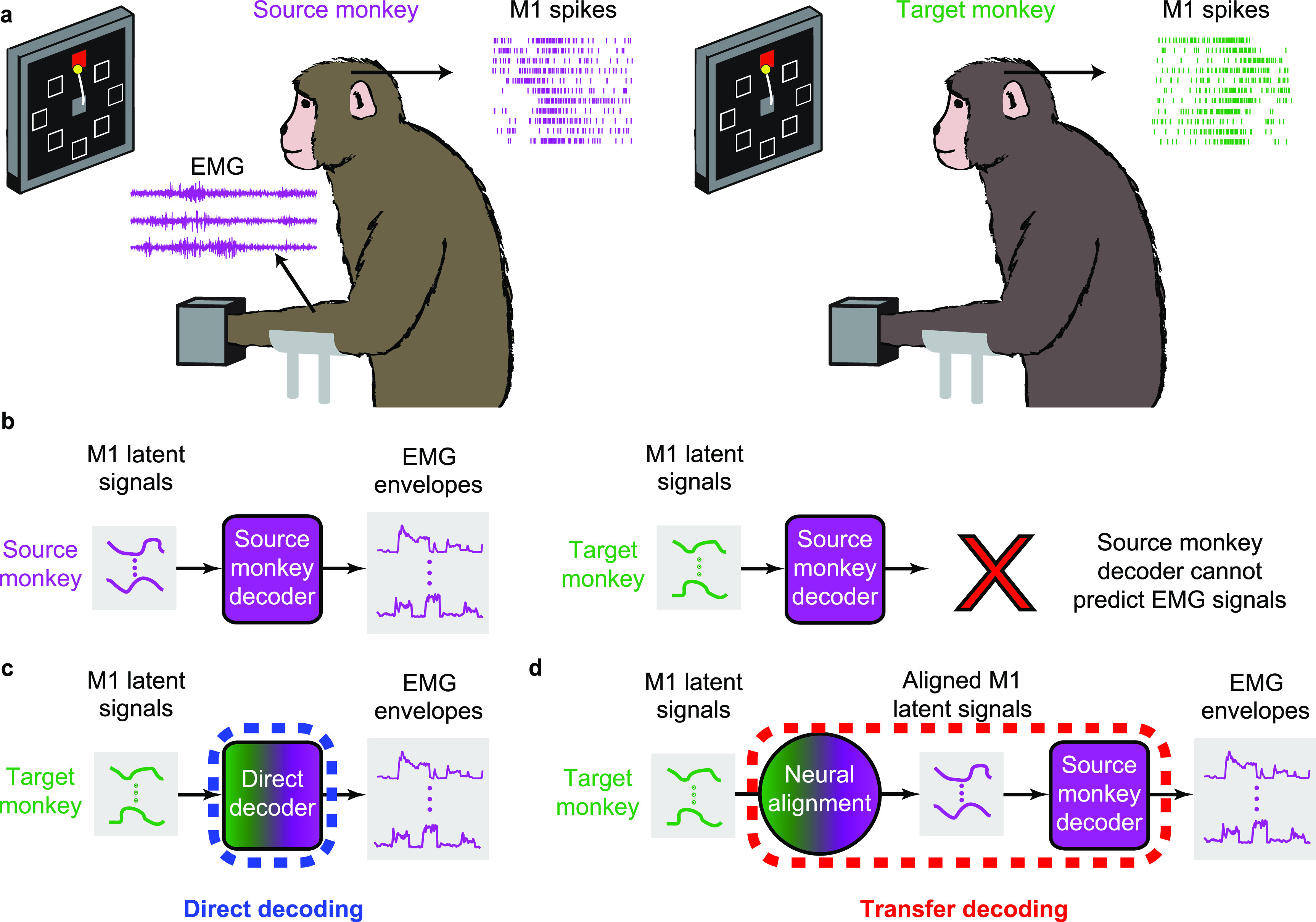
Cross-user decoding of EMG. (a), We recorded neural firing rates from M1 and EMGs from forearm and wrist muscles of a ‘source’ monkey trained to perform the isometric wrist task. We also recorded M1 data from a ‘target’ monkey. (b), The firing rates of the source monkey were projected in a low-dimensional neural manifold and the resulting latent signals used as input to train a source-monkey iBCI decoder. When using the target-monkey M1 data, this decoder fails to accurately predict EMGs. (c), In a first approach, we achieved source monkey EMG predictions by training a decoder between the target monkey M1 signals and the source monkey EMG signals. We call this approach ‘direct decoding’ (d), In a second approach, we computed a decoder solely from the source monkey data, and preprocessed the target M1 latent signals to align them to those of the source monkey. We used the aligned latent signals as input to the source-monkey decoder to obtain predicted EMG. We call this approach ‘transfer decoding’.

We considered data from three different monkeys on four different recording sessions (within a span of 47 d), for a total of 12 sessions. In each session, the monkeys were seated in a primate chair that faced a computer monitor, with their left hand secured within a small box instrumented with a six degree of freedom load cell (JR3 Inc., CA) to measure the forces exerted (figure 1(a)). The load cell measurement axes were aligned with those of the wrist so that flexion/extension forces moved a cursor on the monitor right/left, while radial/ulnar deviation forces moved it up/down. The monkeys were required to move the cursor from a central target towards one of eight peripheral targets uniformly distributed on a circle. A trial started when the monkeys held the cursor in the central target for a random time between 0.2 s and 1.0 s. Then, one of the eight peripheral targets was selected randomly and presented together with an auditory go cue. Monkeys had to reach the outer target within 2.0 s and maintain that force for 0.8 s to receive a liquid reward. In this study, we used data within a window starting 0.5 s before onset of cursor movement and ending 1.0 s after movement onset (see figure S1 for examples of cursor trajectories).

Each monkey was implanted with a 96-channel Utah electrode array (Blackrock Neurotech, Inc.) in the hand area of the right M1, contralateral to the hand used for the task, using standard surgical procedures. In a separate procedure, monkeys were also implanted with intramuscular leads in the forearm and hand muscles of the left arm. The location of each electrode was verified during the surgery by observing the muscle contraction evoked when it was stimulated. Five muscles were implanted in all three monkeys—three major wrist muscles: extensor carpi radialis (ECR) longus, flexor carpi radialis, and flexor carpi ulnaris, and two extrinsic finger muscles: extensor digitorum communis (EDC) radialis and flexor digitorum profundus (FDP). Beside these muscles that were recorded in all three monkeys, we implanted additional muscles in each monkey. These included extensor carpi radialis brevis and extensor carpi ulnaris (ECU) in monkey J, flexor digitorum indicis and opponens digiti minimi and FDP (2 pairs) in monkey S, and ECU, EDC, flexor digitorum superficialis, pronator teres (PT), and supinator (SUP) in monkey K.

We recorded M1 activity using a Cerebus system (Blackrock Neurotech, Inc.). The signals on each channel were sampled at 30 kHz, digitally bandpass filtered (250–5000 Hz) and converted to spike times based on threshold crossings. The threshold on each channel was set with respect to its root-mean square (RMS) amplitude (monkey J: −5.5 × RMS; monkey S: −6.25 × RMS; monkey K: −6.0 × RMS). To extract the smoothed firing rate used in the analysis, we applied a Gaussian kernel (standard deviation of 100 ms) to the spike counts in 20 ms, non-overlapping bins for each channel.

The recorded EMG signals were amplified, bandpass filtered (4-pole, 50–500 Hz), and sampled at 2 kHz. To extract the envelopes used during the analysis, we subsequently rectified and lowpass filtered (4-pole Butterworth, 10 Hz) each EMG channel digitally and subsampled it to 50 Hz to correspond to the bin size of the M1 signals. EMGs were clipped to avoid data points larger than the mean plus 6 times the standard deviation of each channel. We removed the baseline of each EMG channel by subtracting the 2nd percentile of its amplitude. As the more typical normalization to maximum voluntary contraction is not feasible in animals, we instead used the 90th percentile of activation. Both numbers were computed based on the entire experimental session, and we preprocessed the signals for each muscle of source and target monkeys separately.

### 2.2. Human participant task and recordings

The participant, male, 28 years old at the time of the implant, was part of a multi-site clinical trial (NCT01894802) and provided informed consent prior to the experimental procedure. All procedures were carried out in accordance with the ethical standards of the Declaration of Helsinki. He presented with a C5 motor/C6 sensory ASIA B spinal cord injury (SCI) that occurred 10 years prior. He had no spared control of the intrinsic or extrinsic muscles of the right hand but had limited control of wrist flexion and extension. Proximal limb control at the shoulder was intact, as was elbow flexion. However, he had no voluntary control of elbow extension.

We secured a rigid wrist brace to the participant’s right wrist and hand, oriented in a neutral posture on his lap. On each trial, the participant attempted to produce isometric wrist forces in response to the movement of a cursor to eight different radial targets displayed on a screen in front of him. Upward movement corresponded to radial deviation, rightward movements to wrist extension, downward movements to ulnar deviation, and leftward movements to wrist flexion. Diagonal targets corresponded to the appropriate combinations of these gestures. Each trial began with the presentation of the upcoming target. One second after target appearance, a go cue occurred, followed by movement of the cursor to the target (Move: 0.2 s), static hold at the target (Hold: 2.0 s), and return to center (Return: 0.2 s). We instructed the participant to produce step-like force profiles in response to the movement of the cursor. To match the time scale of the monkey data, here we only used data from a 1.5 s window, starting either 0.84 s or 0.78 s before the go cue (see section 3.4 and figure 6(b)).

The participant was implanted with four NeuroPort microelectrode arrays (Blackrock Neurotech, Inc.) in the left hemisphere. Two 96-channel arrays were implanted in the hand and arm areas of M1, while the other two were implanted in somatosensory cortex. In this study, we only used the arrays placed in M1. The signals on each channel were recorded at 30 kHz and high-pass filtered at 750 Hz. Whenever the signal crossed a threshold (−4.25 × RMS), a spiking event was recorded. We used multiunit threshold crossing and considered spike counts in 20 ms non-overlapping bins. We applied a Gaussian kernel (standard deviation of 100 ms) to obtain the smoothed firing rate used in the analysis. For all data analyses, we mirrored the target labels for the human data as his cortical implant is in the opposite hemisphere as that of the monkeys, who used their left hands to perform the task.

### 2.3. Computation and evaluation of EMG decoders

In this study, we tested two cross-user decoding approaches: direct decoding and transfer decoding. Both types of decoders were trained to predict the source monkey’s EMG signals, and used as inputs latent signals computed within a low-dimensional manifold of M1. The direct decoder was trained on target M1 signals (monkey or human; figure 1(c)), whereas the transfer decoding was trained on M1 data from the source monkey (figure 1(d)).

To find the latent signals, we performed dimensionality reduction by applying principal component analysis (PCA) to the M1 firing rates. We set the dimensionality of the latent space to 13, the largest estimate of linear dimensionality across all monkeys and sessions using Parallel Analysis (Altan *et al* 2021). We computed Wiener filter decoders that implemented linear regression to predict the EMG at the current time bin from the latent neural signals stretching to five time bins (100 ms) into the past. The output of the linear filter was then rectified to better match the statistics of the EMG envelopes.

We compared the resulting EMG predictions with the actual EMG recordings of the source monkey. Ideally, the accuracy of the cross-monkey and cross-species decoder as measured by the coefficient of determination (${R^2}$) would approach the performance of the corresponding within-monkey decoder. As the decoded EMGs are multi-dimensional, we computed an ${R^2}$ for each EMG and then reported the average across all muscles, weighted by the variance of each recorded EMG.

### 2.4. CCA alignment of low-dimensional neural signals

The transfer decoder requires that we ‘align’ the latent signals of the target monkey (or human) to those of the source monkey that was used to compute the decoder (figure 1(d)). Similarly, in direct decoding, we tested alignment of the latent signals over time, as a means to address the steady decline in performance of a fixed decoder over time due to the inherent instabilities of the recorded neural signals (Perge *et al* 2013, Sussillo *et al* 2016, Downey *et al* 2018). For both applications, we used Canonical Correlation Analysis (CCA). We labeled the latent neural spaces that we wished to align as ${{\boldsymbol{S}}}$ and ${{\boldsymbol{T}}}$. Both were *M* × *p* matrices, where *M* is the number of samples and *p* the latent dimensionality (*p = 13*). The goal of neural alignment is to make ${{\boldsymbol{T}}}$ as similar as possible to ${{\boldsymbol{S}}}$. When we tested neural alignment with direct decoding, ${{\boldsymbol{S}}}$ and ${{\boldsymbol{T}}}$ were the neural signals associated with the same user but across sessions. In the transfer decoding case, ${{\boldsymbol{S}}}$ and ${{\boldsymbol{T}}}$ were associated with source and target users, respectively.

CCA linearly transforms both ${{\boldsymbol{S}}}$ and ${{\boldsymbol{T}}}$ such that the correlation between ${{{\boldsymbol{C}}}_S}\left( {{\boldsymbol{S}}} \right){{\boldsymbol{\,}}}$ and ${{{\boldsymbol{C}}}_T}\left( {{\boldsymbol{T}}} \right){{\boldsymbol{\,}}}$is maximal, where ${{{\boldsymbol{C}}}_S}$ and ${{{\boldsymbol{C}}}_T}$ are the matrices that implement the linear transformations of the latent signals (figure 3(a)). Since CCA requires a one-to-one correspondence in time between data points, we extracted single trials from the continuous data. We then ordered all trials by target direction and concatenated them prior to applying CCA. Finally, because CCA transforms both sets of signals, we further multiplied ${{{\boldsymbol{C}}}_T}\left( {{\boldsymbol{T}}} \right)$ by the inverse transformation ${{\boldsymbol{C}}}_S^{ - 1}$ to represent ${{\boldsymbol{T}}}$ in the coordinate frame of ${{\boldsymbol{S}}}$. We refer to the resulting latent signals $\overset{\boldsymbol\sim}{\mathbf T\boldsymbol\;}=\;\boldsymbol C_{\boldsymbol S}^{-1}(\;{\boldsymbol C}_{\mathbf T}(\boldsymbol T))$ as the aligned latent signals. For the CCA computation, we used all 13 components of the PCA latent space.

### 2.5. Cross-validation of direct and transfer decoding approaches

For both direct and transfer decoding approaches with the monkey data, we implemented 4-fold cross-validation. We split the data from the source and target monkeys into training and test sets before performing PCA, computing the source-target monkey direct decoder (figure 1(c)), or computing the source-target monkey CCA aligner (figure 1(d)). This resulted in a training set of 96 trials (12 trials and 18 s of data per target direction) and a test set of 32 trials (4 trials and 6 s of data per target direction) for each fold. For the more limited human data, we used only three folds, each consisting of three trials for each of the eight targets. We used all the available data to train the source monkey transfer decoder itself (128 trials in total, 16 trials and 24 s of data per target direction). We opted for using all available data from the source monkey for constructing this decoder as a larger set of movements is typically available for the source monkey compared to the target, and having more data improves the performance of a within-subject BCI decoder.

## 3. Results

### 3.1. Direct mapping between target M1 and source EMG signals enables cross-monkey decoding

Direct decoding offers a simple solution to the problem of mapping neural activity to EMGs in iBCI applications for individuals with a paralyzed arm and hand. Direct decoding establishes a mapping between the neural signals recorded from the iBCI user (the ‘target’) and EMG signals from a ‘source’ individual as they each perform (or attempt to perform) the same movement (figure 1(c)). We began our investigation of direct decoding using monkeys as both source M1 and target EMG inputs to a Wiener filter decoder in an eight-direction wrist force generation task.

**Figure 2.**
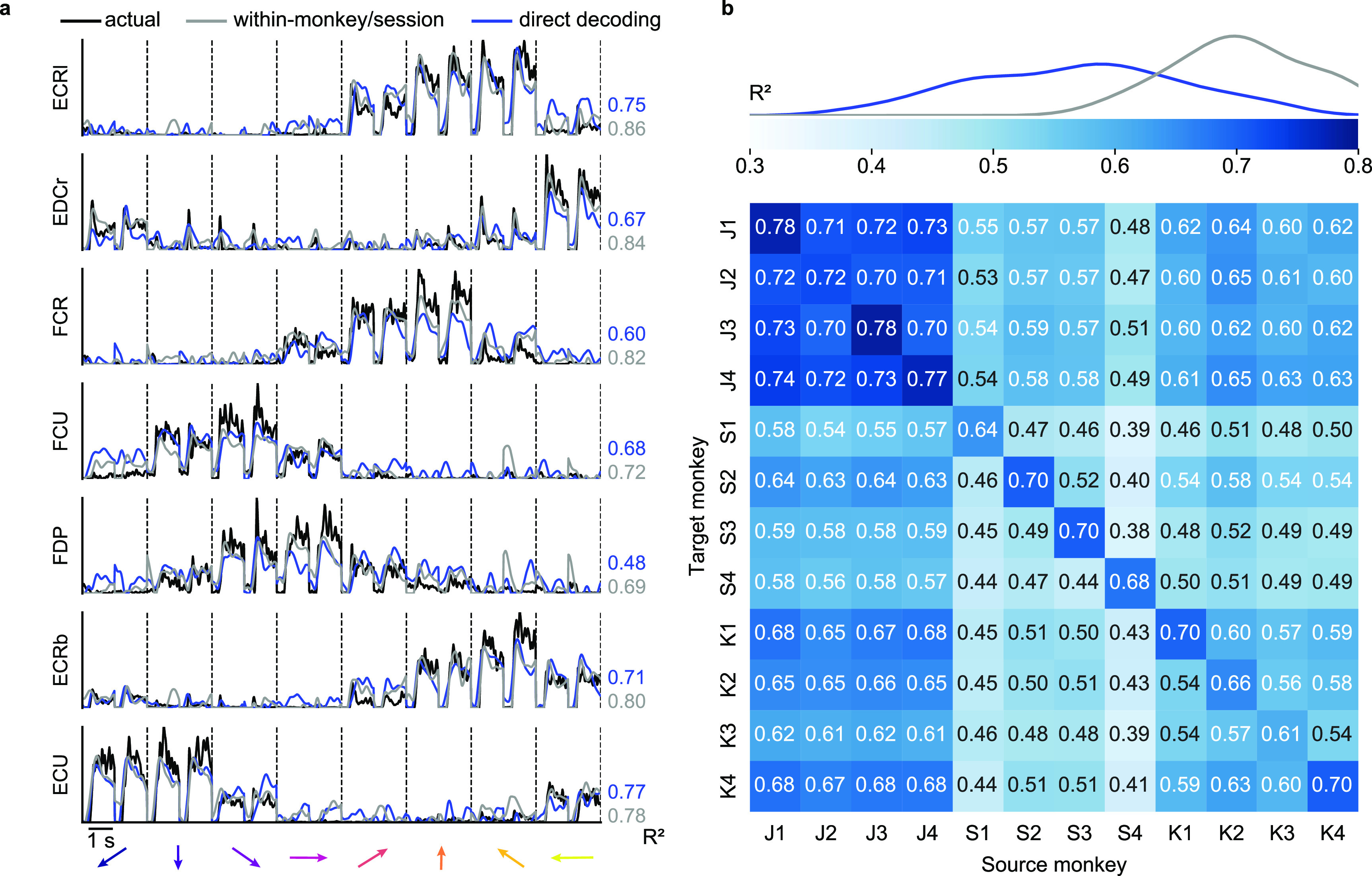
Direct decoding of EMG across monkeys. (a), Cross-monkey EMG predictions obtained with target-monkey latent signals via direct decoder (blue lines). Data for a representative pair of monkeys for two trials in each target direction (directions separated by vertical dashed lines and indicated by arrows at the bottom of each column). These predictions are almost as good as those obtained by using the source-monkey latent signals as input to the decoder (grey lines). The ${R^2}$ values for both within- (grey) and cross- (blue) monkey decoding are shown for each muscle, computed relative to actual EMG recordings of the source monkey (black lines). (b), Overall cross-monkey decoding accuracy (${R^2}$) with direct decoding for all pairs of monkeys. A kernel density estimate plot at the top shows the distribution of cross-monkey (off-diagonal) ${R^2}$ (blue) compared to within-monkey (on-diagonal) ${R^2}$ (grey).

We determined the EMG decoding accuracy by computing the ${R^2}$ between the EMG predictions and the actual EMG signals recorded from the source monkey. Figure 2(a) (blue lines) shows example EMG predictions from a single source-target monkey pair (source monkey: J, first session, target monkey: S, second session). The EMG decoding accuracy ranged between ${R^2}$ = 0.48 and 0.77 for individual muscles. The average EMG decoding accuracy across all muscles, weighted by their individual variances, was 0.64. We compared these to the accuracy of decoders built from the same monkey and session, which ranged between 0.69 and 0.86 for the same muscles, with an average ${R^2}$ of 0.77 (grey lines).

Next, we extended the cross-monkey EMG decoding to all source-target monkey pairs (figure 2(b)). The diagonal entries of the matrices in figure 2(b) show the weighted averages across all muscles of the within-monkey decoding accuracy for each session. The off-diagonal elements represent either cross-monkey or within-monkey/cross-session accuracy. We included the within monkey/cross-session decoding accuracy as a more realistic comparison with the cross-monkey (and implicitly cross-session) analyses. The curves at the top of the panel indicate the kernel density estimate of the distributions for the off-diagonal and the on-diagonal accuracies. On average, cross-monkey direct decoding retained 80% of the accuracy of within-monkey decoding and 97% of the within-monkey/cross-session accuracy.

### 3.2. Latent signals across monkeys become similar after neural alignment

An alternative approach to obtain EMG predictions for individuals with paralysis is to use a fixed decoder trained on a source monkey from which both neural and EMG activity can be recorded, and ‘transfer’ it for use with the M1 signals recorded from the target iBCI user. A decoder using neural firing rates as inputs cannot be used directly for another individual as there is no correspondence between neurons recorded from different subjects. Instead, we compute decoders based on inputs from low-dimensional latent signals extracted from the firing rates. An essential step is to align the latent signals of the target iBCI user to those of the source monkey (figure 1(d)) (Farshchian *et al* 2019, Degenhart *et al* 2020, Gallego *et al* 2020, Ma *et al* 2023).

**Figure 3.**
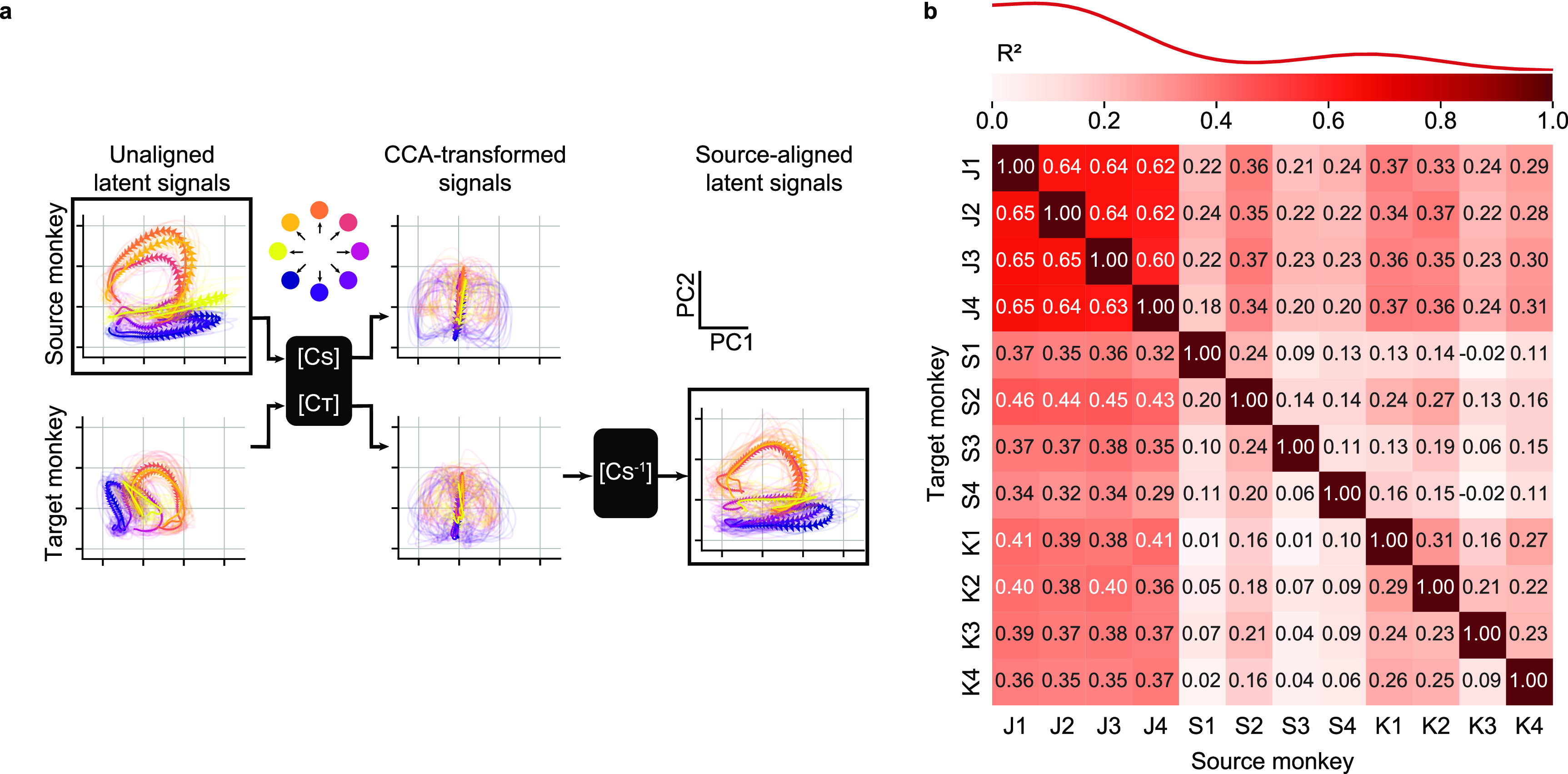
Latent neural signals become more similar across monkeys after neural alignment. (a), Representative latent signals described by the first two principal components for a source (top left) and target (bottom left) monkeys. We used CCA to transform the latent signals such that they were maximally correlated (center). The CCA-transformed signals are more similar, but neither looks like the original source-monkey trajectories. Consequently, we further transformed the target-monkey latent signals in the CCA aligned space by the using the inverse of the source-monkey CCA transformation ($C_S^{ - 1}$) to map them back into the original source-monkey latent coordinates (bottom right). Data were averaged across all trials for each target direction; single trial trajectories are shown as lighter traces. Arrows indicate the temporal evolution of the trajectories (from 0.5 s before to 1 s after cursor movement onset). (b), Overall cross-monkey latent signals similarity (${R^2}$) after CCA alignment for all pairs of monkeys. A kernel density estimate plot shows the distribution of cross-monkey latent signals ${R^2}$ similarity after CCA alignment.

First, we used data from three monkeys to test the extent to which we could align the latent neural signals collected from a source monkey to those of a target monkey. Figure 3(a), left column shows an example of the latent signals obtained by projecting the neural activity of the source and target monkeys into their corresponding neural manifolds (source monkey: J, first session, target monkey: S, second session). The unaligned latent signals of the two monkeys are similar, in that the trajectories corresponding to each of the eight targets are well-separated and traverse roughly parallel paths through their respective latent spaces. However, despite their similar shapes, the two sets of latent signals differ in scale and orientation.

We next used CCA to linearly transform both sets of low-dimensional signals such that they were maximally correlated (figure 3(a), middle column). The transformed sets of latent signals traverse very similar regions of the new (transformed) latent space, but neither looks like the original source monkey trajectories, as both were transformed by CCA. Consequently, we further processed the transformed target monkey latent signals by using the inverse of the canonical correlation transformation from the source monkey. This two-step process allowed us to align the target monkey signals to those of the source monkey (figure 3(a), right column), and use these as input to the decoder that had previously been trained on data from the source monkey. The aligned trajectories for the two monkeys (figure 3(a), upper left, lower right) are a remarkably close match.

We computed the ${R^2}$ between source monkey latent neural signals and CCA-aligned target monkey latent neural signals for the entire dataset comprising four sessions for each of three monkeys (figure 3(b)). CCA alignment significantly improved the similarity of all source/target monkey latent signal pairs, both within-monkey/cross-session (${R^2}$ before alignment: −0.37 ± 0.11; after alignment: 0.34 ± 0.04 (mean ± s.e.); *P* ∼ 0, Wilcoxon’s signed rank test) and cross-monkey $({R^2}$ before alignment: −1.08 ± 0.08; after alignment: 0.25 ± 0.01 (mean ± s.e.); *P* ∼ 0, Wilcoxon’s signed rank test).

### 3.3. Neural alignment allows to transfer EMG decoder across monkeys

Next, we evaluated the performance of the transfer decoding approach using the CCA aligned latent signals as inputs to the decoder. Just as we did for direct decoding, we quantified EMG decoding accuracy by computing the ${R^2}$ between the EMG predictions and the actual EMG traces of the source monkey.

**Figure 4.**
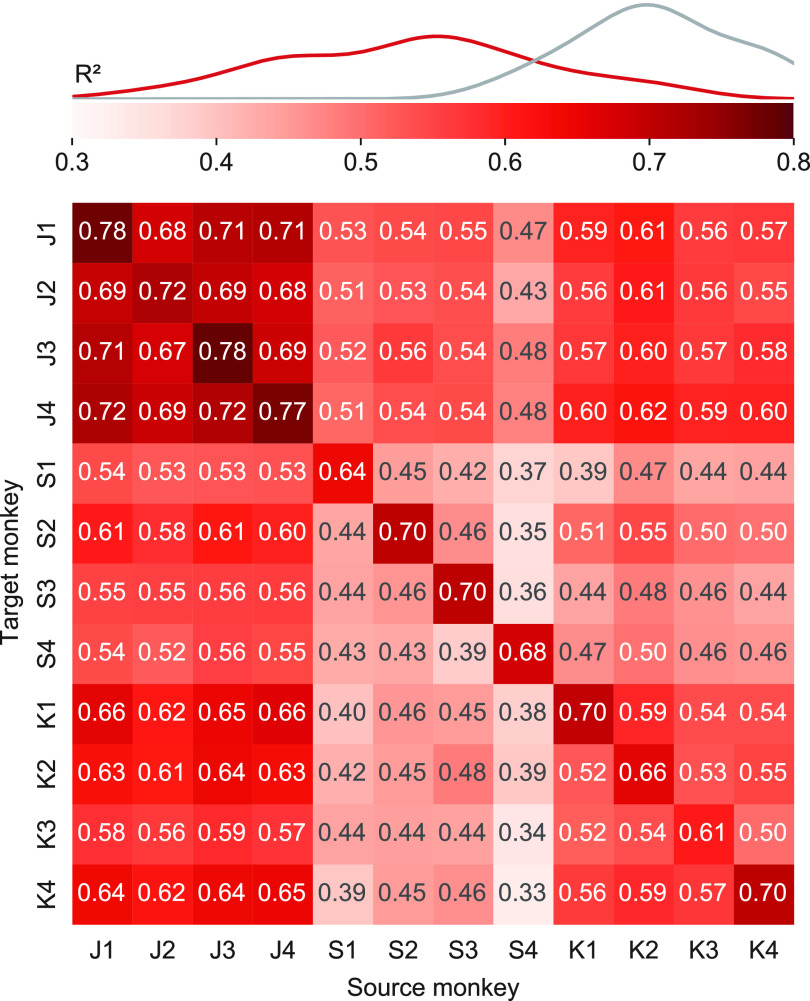
Transfer decoding of EMG across monkeys. Overall cross-monkey decoding accuracy (${R^2}$) with transfer decoding for all pairs of monkeys. A kernel density estimate plot shows the distribution of cross-monkey ${R^2}$ (red) compared to within-monkey ${R^2}$ (grey) as in figure 2

Transfer decoding results are summarized in figure 4 for all source-target monkey pairs. The average cross-monkey EMG decoding accuracy was 0.53, virtually identical to the average within-monkey/cross-session accuracy of 0.55. Across all individuals and datasets, cross-monkey transfer decoding retained 95% of the within-monkey/cross-session accuracy, and even 75% of the within-monkey/within-session decoding accuracy.

**Figure 5.**
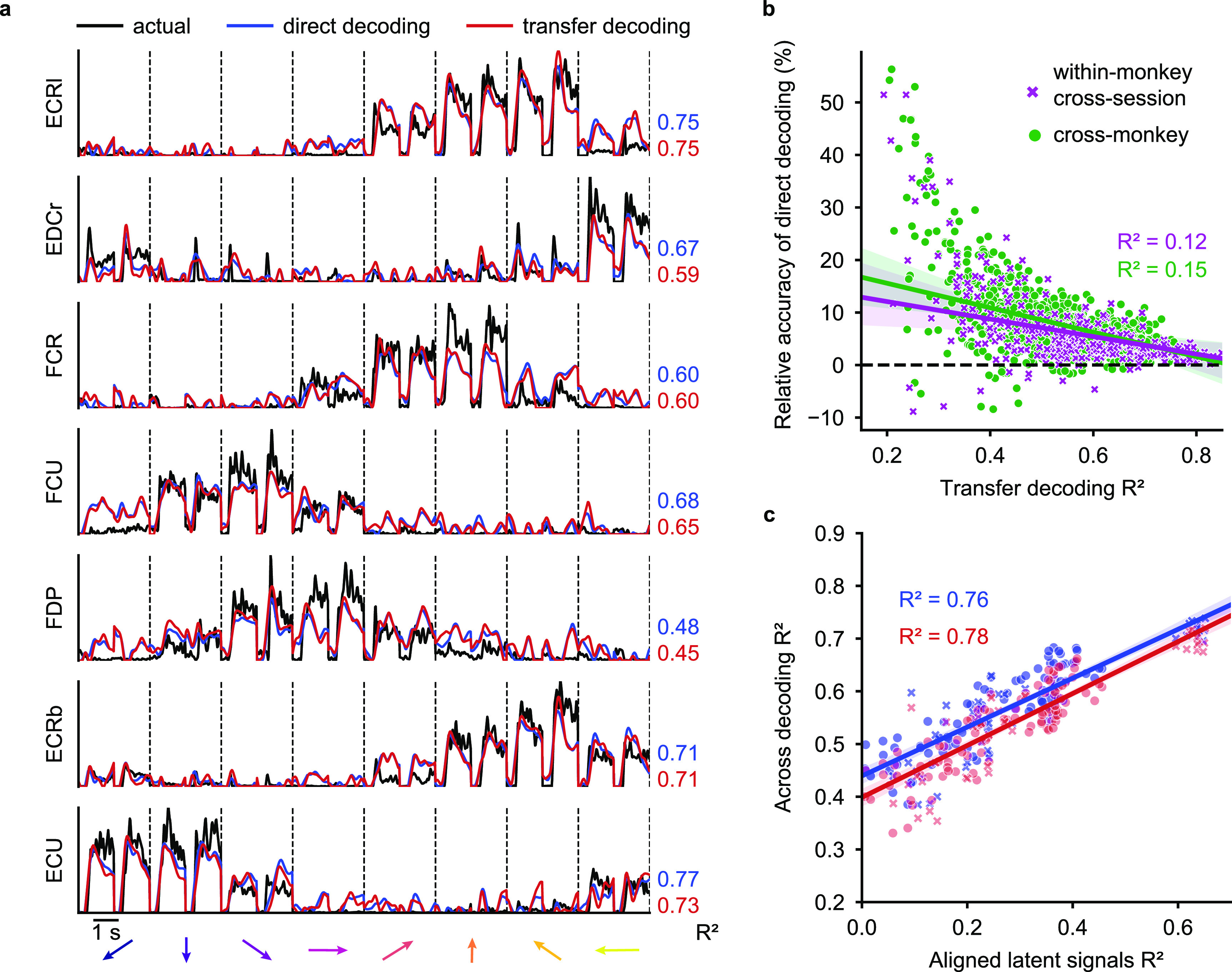
Comparing direct decoding and transfer decoding. (a), Representative cross-monkey EMG predictions obtained with direct (blue lines) and transfer decoding (red lines) compared to source monkey ground truth (black lines, as in figure 2(a)). (b), Element-by-element scatter plot comparing direct and transfer decoding performance. Each point represents the within-monkey/cross-session (purple) or cross-monkey (green) accuracy of transfer decoding (*x*-axis) versus the relative accuracy of direct decoding (*y*-axis) of a single muscle. Direct decoding generally yielded higher decoding accuracy for both cross-monkey and cross-session predictions, especially when decoding accuracy was low. (c), Element-by-element scatter plot for the matrices in figures 4 and 2(b). Latent signals of monkey pairs with higher similarity after CCA alignment yielded higher decoding accuracy for both cross-monkey (circle dots) and within-monkey/cross-session (x signs) predictions.

Figure 5(a) shows example EMG predictions for both the direct and transfer approaches, as well as the actual EMGs of the source monkey for the same source-target monkey pair shown in figures 2 and 3. Although the accuracy of the two methods was quite similar, that of direct decoding was slightly higher.

We quantified transfer decoder performance for all monkey pairs in figure 5. Each point in the scatter plot shows the ${R^2}$ between actual and decoder-predicted EMG signals for an individual muscle for both methods. For both the cross-monkey (figure 5(b), green symbols) and cross-session (purple symbols) scenarios, the direct decoding yielded a consistently higher prediction accuracy ${R^2}$, although the magnitude of this difference is small compared to the range of observed ${R^2}$ values.

The similarity between the EMG decoding accuracy matrices in figures 2(a) and 4(a) and the neural alignment accuracy matrix in figure 3(b) suggests that accuracy of decoder performance with either direct or transfer decoding is dependent on successful neural alignment. We quantified the relationship by constructing an element-by-element scatter plot of the decoding and alignment matrices (figure 5(c)). Indeed, both the cross-monkey (green) and cross-session (purple) decoding accuracy was well predicted by the success of the CCA alignment, with an ${R^2}$ of 0.76 for direct and 0.78 for transfer decoding across all datasets.

### 3.4. Monkey to human EMG decoding

Having demonstrated that cross-monkey EMG decoding is possible, we asked whether the same two approaches could be implemented for cross-species decoding from a monkey to a human. We thus tried to either train a direct decoder or transfer a monkey decoder to predict EMG signals from the neural activity of a human with paralysis. For this analysis, we used the first session from monkey J (dataset J1) as the source monkey, as it had the highest within-session decoder performance among all datasets.

We performed these experiments with one participant who had a partial SCI at C5/C6, and was implanted with two 96-channel electrode arrays, one each in the arm and hand areas of the left primary motor cortex (M1). We recorded multi-unit spiking activity as the participant, who had some remaining ability to produce wrist extension forces, attempted to produce isometric wrist forces in eight radial directions. Cursor control and task design were essentially the same as in the monkey experiments, with two exceptions. First, the participant attempted to use his right arm and hand (contralateral to the arrays), thereby reversing, in world coordinates, the flexion and extension muscle activity relative to those of the monkey. Consequently, for direct decoder and CCA alignment computation, we mirrored the target directions for the human data about the vertical plane containing the up/down directions of force exertion. Second, cursor movement was not under the control of the participant, but instead occurred automatically as in the standard approach to ‘observation’ decoder training (Hochberg *et al* 2006, Willett *et al* 2019). One second after target appearance, a go cue occurred and the cursor moved to the target in 0.2 s, where it remained for 2.0 s before returning to the center in 0.2 s. We instructed the participant to attempt to produce the forces necessary to control the position of the cursor.

**Figure 6.**
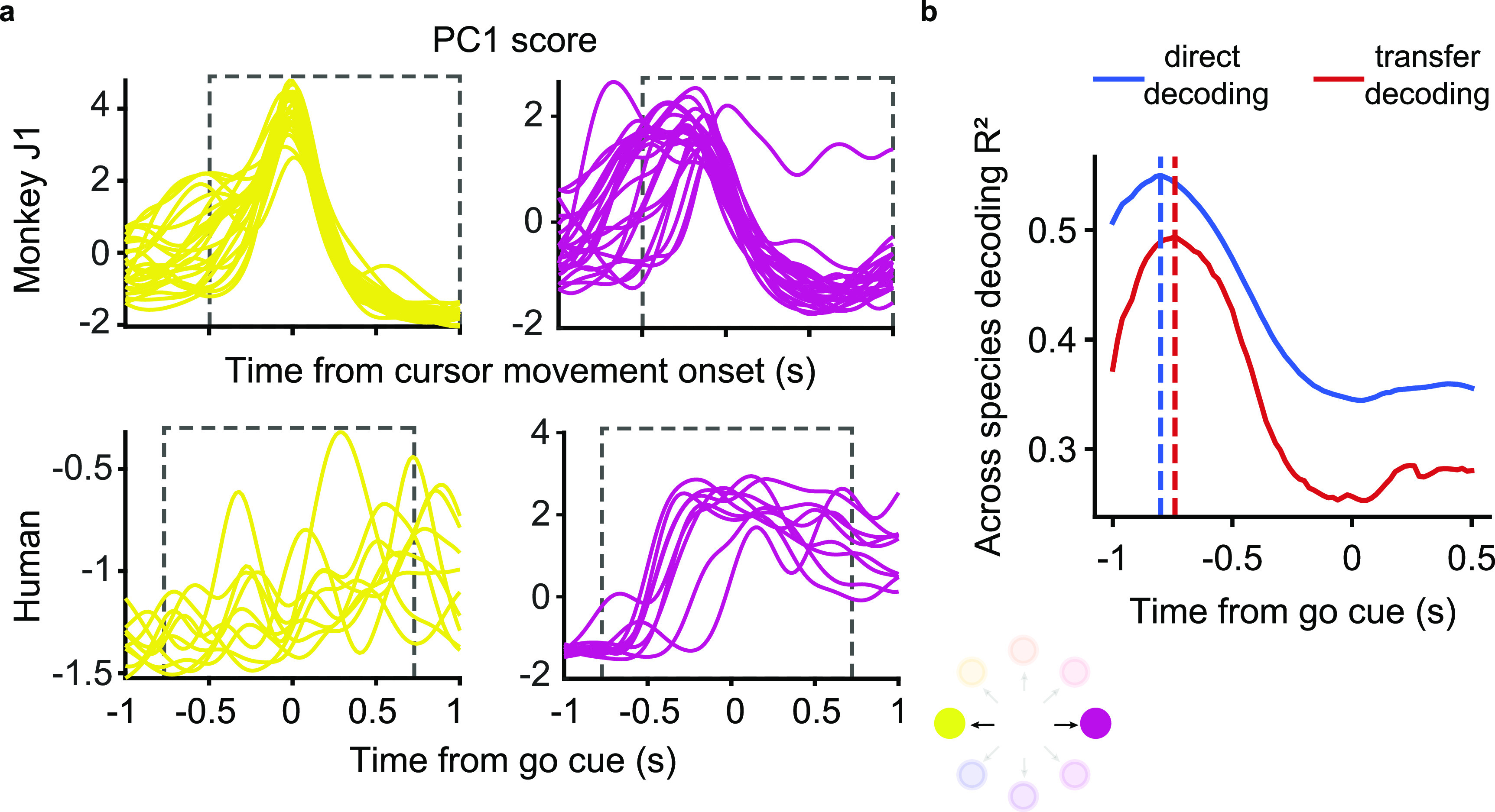
Accuracy of monkey-to-human EMG decoding depends on the latency relative to the go cue used for human trial segmentation. (a), Single-trial M1 data from the source monkey (top, first session from monkey J) and the human participant (bottom) projected onto the first principal component (computed in each case using the entire corresponding dataset) for a pair of oppositely directed targets (left: yellow, right: purple). The neural responses recorded during the human’s attempted task had greater trial-by-trial variation in timing and magnitude compared to those of the monkey. (b), EMG decoding accuracy with direct (red) and transfer decoding (blue) as a function of the time index relative to the go cue used to segment the human trials. The vertical dashed line indicates the time at which greatest EMG decoding accuracy is achieved; note that it precedes the go cue.

Figure 6(a) shows the time course of the neural activity of the hand M1 array projected onto its first principal component, during trials corresponding to two oppositely directed targets. The human data is more variable across trials than that of the monkey, both in onset timing and in magnitude; this is likely due to the lack of actual force and force-related feedback, or because participant’s M1 has become ‘out of practice’ in generating motor activity. To minimize the effect of timing variability on the cross-species decoding process, we tested a range of time offsets between neural activity and target EMG when selecting the neural data to use as input to our two decoders. We computed the EMG decoding ${R^2}$ for each tested offset, and report the ${R^2}$ for the best-performing offset. Under these conditions, we achieved maximal decoding accuracy when aligning the human trials 0.84 s and 0.78 s prior to the go cue (figure 6(b)) for direct and transfer decoding respectively.

**Figure 7.**
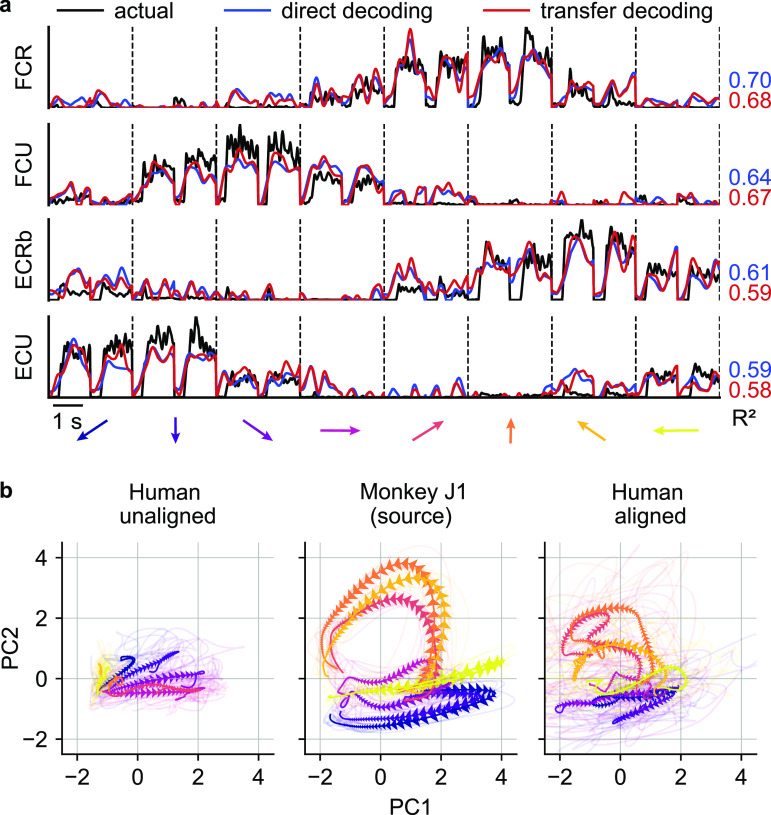
EMG decoding from a human with tetraplegia. (a), EMG predictions for the four major wrist muscles obtained via direct (blue lines) and transfer decoding (red lines). Actual EMG recordings of the source monkey (black) provide a ground truth. (b), Latent M1 trajectories described by the first two principal components of the M1 data (left) recorded as the participant attempted to perform a wrist isometric task, and those for the source monkey (center; first session from monkey J). The human neural recordings had a stereotypical low-dimensional structure for each of the eight target directions, with large inter-trial variability (shown by the lighter traces). Despite this increased variability, CCA alignment recovered a shape with better similarity to that of the source monkey’s latent signals (right).

Given this optimal time-alignment, the average ${R^2}$ between predicted and actual EMG using the direct decoding approach was 0.55 (figure 7(a), blue lines), which is 98% of the performance of the corresponding cross-monkey predictions, and even 78% of the average within-monkey/within-session decoding. The transfer decoding approach achieved similar accuracy, with an average ${R^2}$ of 0.49 (figure 7(a), red lines).

Despite the greater inter-trial variability of the human recordings, the latent signals of the human neural activity traverse well-separated, stereotyped trajectories for each of the eight targets (figure 7(b), left panel). CCA alignment allowed us to match these latent signals to those of a target monkey, although degree of alignment was lower than for typical pairs of monkeys (figure 7(b), middle and right panels; ${R^2}$ increased from −0.56 to 0.10).

### 3.5. Decoders need to be stabilized in the face of changes in the neurons that are recorded over time

**Figure 8.**
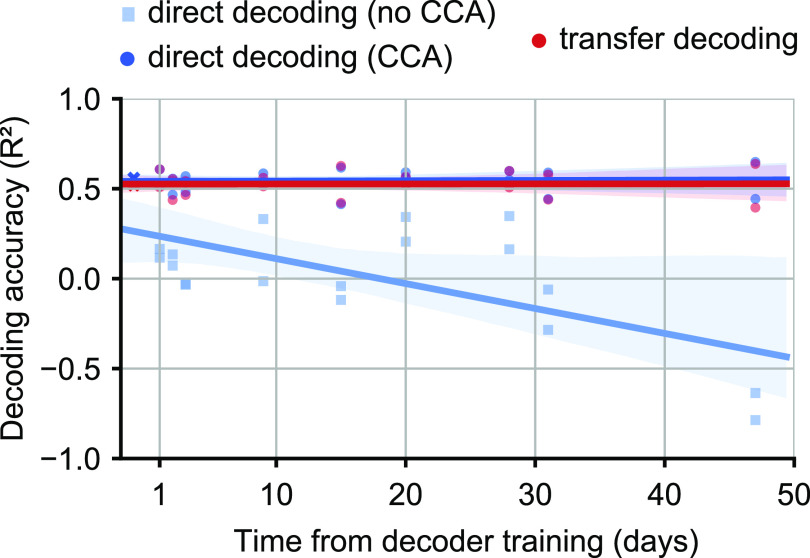
A fixed direct decoder needs to be aligned across time. (a), EMG prediction accuracy over time using a fixed ‘day-0’ direct decoder trained on the first target monkey session. We compared the performance of the fixed direct decoder before (turquoise) and after (blue) within-monkey/across-time CCA alignment between the day-k and day-0 target neural data. CCA alignment stabilized the performance of the fixed direct decoder over time, such that it achieved performance similar to that of the transfer decoding approach (red).

The performance of any fixed iBCI decoder tends to decline over time due to the inherent instabilities of the recorded neural signals (Perge *et al* 2013, Sussillo *et al* 2016, Downey *et al* 2018). One simple solution to counteract this effect is to recompute the iBCI decoder at each session, as we have done with the direct decoding approach. Alternatively, various groups have proposed the use of neural aligners that match the statistics of neural recordings from a later day (‘day-k’) to those from the day when the decoder was calibrated (‘day-0’) (Farshchian *et al* 2019, Degenhart *et al* 2020, Gallego *et al* 2020, Karpowicz *et al* 2022, Ma *et al* 2023). These neural alignment approaches were adopted to avoid the scenario in which an iBCI user would need to relearn the dynamics of a decoder recomputed each session. In the transfer decoding approach, CCA alignment works implicitly across time as well as across users, thereby automatically addressing this problem. However, this is not the case for direct decoding; its accuracy was maintained only because we trained a new decoder for every session. If instead the direct decoder is fixed, its performance rapidly declines with time, as expected (figure 8, blue squares). Therefore, we investigated the effectiveness of using CCA to align the day-k and day-0 latent signals using monkey data (figure 8, blue dots and line). In this scenario, the greater direct decoding accuracy over the transfer decoding approach disappears (red and blue circles).

### 3.6. Task generalization of cross-individual EMG decoding

Beyond the question of how well a given decoder performs when trained and tested on similar movements, is the question of how well an EMG decoder can extrapolate to different movements. This question may be particularly important for the cross-individual decoders we are developing, as they depend on the two subjects performing similar movements.

**Figure 9.**
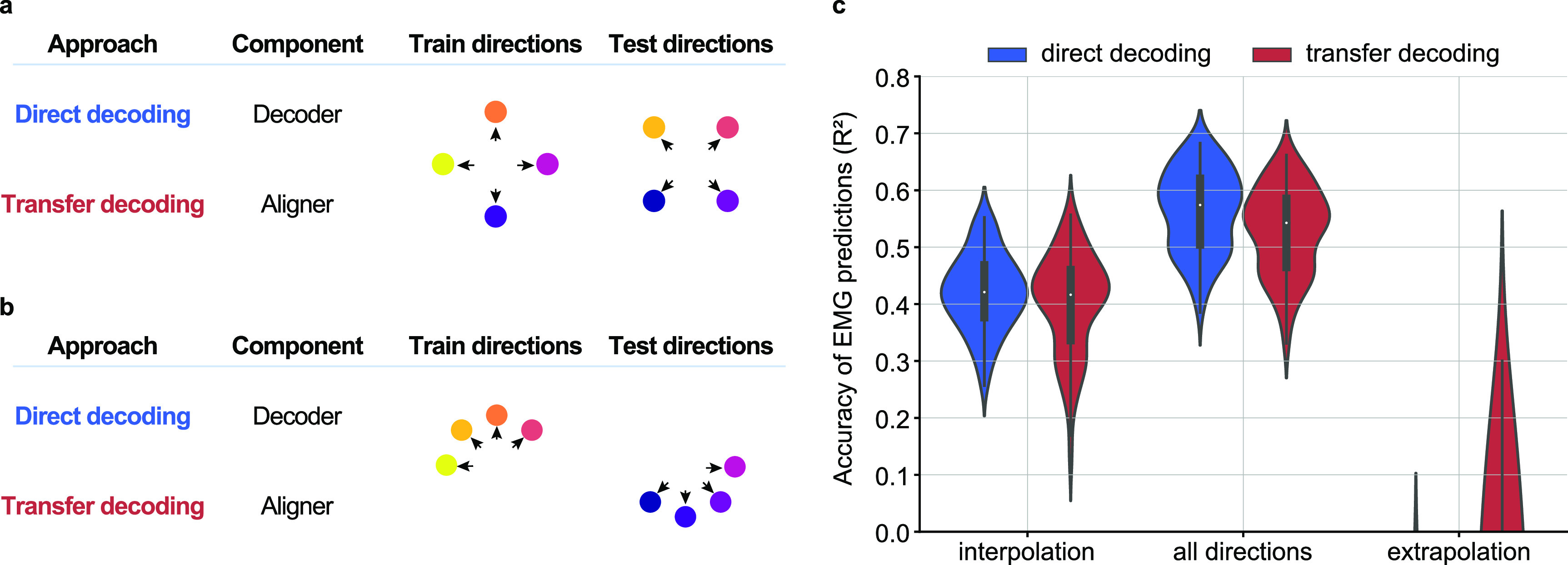
Task generalization of cross-monkey EMG decoding. Generalizability of the direct (blue) and transfer (red) decoding was assessed by training either the direct decoder or the transfer decoder using only a subset of all eight movement directions. (a), Task generalization when *interpolating* (i.e. training on cardinal directions and testing on diagonal directions); (b), Task generalization when *extrapolating* (i.e. training on adjacent upper directions and testing on lower directions). (c), Violin plots for the overall cross-monkey decoding accuracy for all pairs of monkeys when training and testing on all target directions (center), when interpolating (left), and when extrapolating (right). When interpolating, cross-monkey decoding is still possible with both direct and transfer decoding, albeit with lower accuracy. When extrapolating, the cross-monkey decoding generally failed, as indicated by the negative ${{{{R}}}^2}$ values.

Using exclusively the monkey datasets, we tested the task generalization performance for both cross-individual decoding approaches. We first investigated decoder performance on test targets that interpolated the four cardinal directions used for training (figure 9(a)). We also tested an extrapolation condition in which we trained on the four lower targets and tested on the upper targets (figure 9(b)). For the three conditions (full training set, interpolated training data, extrapolated training data), the direct decoding approach achieved an ${R^2}$ of 0.56, 0.42, and −0.45, respectively (figure 9(b), red). In contrast, the transfer decoding approach achieved an ${R^2}$ of 0.53, 0.40, and −0.23, respectively (figure 9(b), blue). Direct decoding significantly outperformed transfer decoding both when using the full training set and in the interpolation case (*P* ∼ 0, Wilcoxon’s signed rank test). Neither approach worked well for the extrapolation case, as the average ${R^2}$ was negative for both.

## 4. Discussion

When surveyed, nearly 80% of the individuals with high-level SCI would elect brain surgery to recover some control of their own hands (Blabe *et al* 2015). However, the ‘observation based’ decoders currently used to map motor cortical activity to movement kinematics cannot be used for prediction or control of an unobservable motor output, such as muscle activity. Here, we tested two possible solutions to this problem. The first is the ‘direct decoding’ approach, in which we mapped the neural signals recorded from an iBCI user to muscle activity of another individual (or monkey) performing the same movements. In the second approach, which we call ‘transfer decoding’, we used a decoder trained on both M1 and EMG signals from a ‘source’ monkey and then transferred it to another monkey or to a human iBCI user. Transfer is achieved by aligning the low-dimensional latent neural signals from that user to those of the source monkey for which the decoder was computed. We investigated the performance of both approaches for cross-individual transfer both from one monkey to another and from a monkey to a human with quadriplegia.

The two approaches yielded similar EMG prediction accuracy, although direct decoding was on average 5% more accurate provided the decoder was retrained at each session. However, when we held the direct decoder fixed and fed it with latent signal inputs that were aligned between sessions with CCA, there was no difference between the two approaches. Finally, we showed that while both approaches were able to generalize to a set of interpolated targets, neither could extrapolate to test targets in a completely different part of the workspace.

Overall, our work indicates that cross-user EMG decoding is possible even between monkeys and humans. This would allow the use of predicted EMG to control the FES of muscles to restore voluntary arm movement or movement of an anthropomorphic limb actuated with control properties designed to mimic those of the musculoskeletal system.

### 4.1. Clinical applications of a monkey-to-human biomimetic decoder

Existing iBCIs have allowed paralyzed individuals to control a computer cursor in two dimensions with only a few minutes of practice (Hochberg *et al* 2006, Brandman *et al* 2018), to control a three-dimensional robot end-effector (Hochberg *et al* 2012) within the first few hours of training (Collinger *et al* 2013) and eventually over months, even systems with seven (Collinger *et al* 2013) or ten degrees of freedom (Wodlinger *et al* 2014). However, these kinematic iBCIs present the user with an entirely different physical plant, one without mass or other dynamics, and no direct means to control applied forces. Limited prior attempts to incorporate dynamics by combining position and joint torque control for 2D planar reaching have proven difficult to control (Fagg *et al* 2009, Chhatbar and Francis 2013), despite the well documented evidence that force and muscle-like information is encoded in neurons in the primary motor cortex (Evarts 1968, Cheney and Fetz 1980, Kalaska and Hyde 1985, Maier *et al* 1993, Hepp-Reymond *et al* 1994, Lemon *et al* 1995, Holdefer and Miller 2002, Sergio and Kalaska 2003, Morrow *et al* 2007, Oby *et al* 2013).

The mammalian neuromuscular system controls the motion of the arm and digits, the stiffness of joints, and exerted forces, all through the modulation of muscle activity. One approach to a more biomimetic form of BCI might be to use the predicted EMG signals to control muscle force directly, through the electrical stimulation of the muscles or peripheral nerves. This technique, known as FES, is used to improve stance and walking (Granat *et al* 1993, Thrasher *et al* 2006, Daly *et al* 2011) as well as grasping (Peckham *et al* 1980, Snoek *et al* 2000, Peckham *et al* 2001, Popovic *et al* 2002) following stroke or SCI. Patients with C5-C6 SCI were able to use their preserved voluntary shoulder movements to trigger preprogrammed stimulation and regain some ability to grasp objects (Taylor *et al* 2002b). This approach becomes much more limited in patients with higher level cervical SCI, as those needing the greatest restored movement have the least available peripheral control signals. iBCIs offer an alternative solution by providing the means to obtain more natural, higher-dimensional control signals than those derived from residual movements.

Our group previously designed an iBCI-controlled FES system that enabled monkeys with temporary paralysis of the hand muscles induced by a peripheral nerve block to perform hand movements; decoded EMGs were used to modulate stimulation of five electrodes implanted in different compartments of three hand flexor muscles (Ethier *et al* 2012).

Two other groups have developed brain-controlled FES systems in which human participants with cervical SCI were able to control simple elbow, wrist, or hand movements. One approach used six parallel decoders, each predicting one of six wrist or finger movements. The decoder with the highest confidence triggered a muscle stimulation pattern consisting of at most three intensity levels, designed to approximate the decoded movement (Bouton *et al* 2016). In another approach, real-time velocities of the elbow, wrist, hand, and shoulder were decoded from M1, and controlled in a feedback system using a combination of muscle and nerve stimulation (Ajiboye *et al* 2017).

While promising as additional proofs of concept, these approaches achieved only limited control of a small number of dimensions, using decoders that were not based on the brain’s normal control of movement, which we attempted to mimic by directly inferring muscle activity from M1. Here, we show two possible approaches to mapping the motor cortical activity of a person with paralysis into intramuscular EMG signals that resemble those of a monkey performing a similar task.

It must be acknowledged that significant complexity remains in using FES to execute movement even with perfectly inferred patterns of muscle activity. While surface stimulation can be used, it has limited spatial selectivity and generates considerably larger artifacts that interfere with M1 recordings. Implanted electrodes would very likely include a combination of intramuscular and nerve cuff electrodes, the latter being the only realistic way of accessing large numbers of muscles, particularly intrinsic hand muscles (Memberg *et al* 2014). Spinal cord stimulation may also be important (Saigal *et al* 2004, Barra *et al* 2022, Powell *et al* 2023), as it avoids the problem of fatigue (Collins 2007) that is caused by electrical neuromuscular stimulation. Despite its challenges, recent surveys indicate that a large majority of individuals with high-level cervical SCI would be willing to undergo brain surgery to restore hand functions (Blabe *et al* 2015), suggesting a willingness also for surgical implantation of FES electrodes.

Given good EMG inference and effective FES, the redundancy of the hand musculature which allows most actions to be produced through a variety of different patterns of muscle activity (Bernstein 1966, Santello and Soechting 2000, d’Avella *et al* 2003) presents an ironic problem. In our monkeys, EMGs were consistent across time for a given monkey (figure S2), but less so across monkeys (figure S3). As the muscle activity patterns became more dissimilar across monkeys, so did their latent neural representations (figure S5(d)). Even more so, the human participant may have intended to use a somewhat different pattern of EMG from that of the source monkey. However, to the extent that the biomechanics of the human hand resembles that of the monkey, the resultant EMG predictions should be biomechanically appropriate to accomplish the task as the source monkey did. The discrepancy would impose an interesting motor-adaptation problem as the user learns to interact with the decoder. An analogous ‘virtual surgery’ paradigm has been used to study the role of synergies in motor control (Berger *et al* 2013); this process could inform future muscle-based decoder design.

In the face of these challenges, we are also working to implement control via a musculoskeletal model of the hand that would work independently of FES (McFarland *et al* 2023). Such control could be implemented initially in VR, using a physics engine like Mujoco (Todorov *et al* 2012), before ultimately being used to control a robotic prosthesis. In principle, this approach will allow control not only of joint kinematics, but also contact forces and joint impedance, as in the actual hand (Blana *et al* 2020).

Whether movements would be actuated via FES or a musculoskeletal model, there is the additional problem of not being able to record all the target muscle EMGs for our training data, whether in the context of direct of transfer decoding model. A musculoskeletal model also gives us a potential solution to this problem through the use of computed muscle control (CMC) (Thelen *et al* 2003). CMC will allow us both to fine-tune the musculoskeletal model, as well as to infer the activity of muscles we did not record from.

### 4.2. Neural representations of motor intent are similar across monkeys and humans

The neural population activity in many brain areas is constrained to a low-dimensional neural manifold. Furthermore, there is increasing evidence that the dynamics of specific patterns of activity within the manifold, the latent signals, underlie the computations required for planning and executing movements (Mazor and Laurent 2005, Mante *et al* 2013, Cunningham and Yu 2014, Gao and Ganguli 2015, Elsayed and Cunningham 2017, Gallego *et al* 2017, Williamson *et al* 2019). In the primary motor cortex, recorded latent signals that differ across days can be mathematically transformed (‘aligned’) to be more similar to each other. These aligned latent signals maintain a remarkably stable relation to behavior over months and even years (Gallego *et al* 2020). As a consequence, a fixed decoder that uses these aligned signals as inputs remains accurate across long periods without supervised recalibration (Degenhart *et al* 2020, Gallego *et al* 2020, Karpowicz *et al* 2022, Ma *et al* 2023).

Manifolds transcend the analysis of individual neurons, and make the comparisons of population activity across individuals more readily interpretable (Dabagia *et al* 2023). Recently, Safaie *et al* used similar alignment techniques to show that latent signals in M1 were preserved across monkeys as they performed a center-out reaching task using a planar manipulandum; latent signals were also preserved in the dorsolateral striatum of mice that grasped and pulled a joystick (Safaie *et al* 2022). Other recent studies revealed that low-dimensional neural structure within the hippocampus and the sensorimotor cortex is preserved across rats for a variety of behaviors, ranging from locomotion along a linear track inside a maze to unconstrained movement in an arena (Rubin *et al* 2019, Chen *et al* 2021, Nieh *et al* 2021, Melbaum *et al* 2022).

Not only was the neural representation of the isometric wrist task similar across monkeys, but also the similarity extended even to a paralyzed human attempting to perform the same task. The preserved motor intent signals between individuals enabled successful monkey-to-human EMG decoding, even though the decoding accuracy was not as high as when using M1 data from a target monkey. The lower cross-user decoding accuracy from the human M1 data could be attributed to the greater inter-trial variability of the corresponding latent trajectories compared to those of the monkeys (figure 6(a)). The increased trial-to-trial variability partly resulted from the absence of any actual force or force-related feedback. Moreover, the variability was influenced by the participant’s tendency to anticipate the go cue during the attempted movements (figure 6(a)). As a result, the accuracy of both direct decoding and transfer decoding peaked for time alignment *prior to* the go cue (figure 6(b)). Remarkably, while these factors could have had a detrimental impact on decoding accuracy, the EMG predictions from either cross-user decoding approach with human M1 data still resembled the actual EMGs of the source monkey.

### 4.3. Comparison of direct and transfer decoding

Direct decoding was simple and consistently outperformed transfer decoding by a small amount, as long as the decoder was retrained each session. Unlike transfer decoding, which requires that both M1 and EMG data be collected from the source monkey, direct decoding has the advantage that it would be possible to use EMGs collected from able-bodied humans instead of monkeys, which would reduce any concern about the similarity of monkey and human biomechanics.

The task we studied here was highly stereotypic and allowed for individual trials to be aligned in time, a requirement for both direct decoding and the CCA alignment used in transfer decoding. This limitation is fundamental for direct decoding; however, CCA could, in principle, be replaced with an unsupervised method that aligns the statistics of clouds of points independently of the time course of the associated signals. Such approaches have been used to align neural signals from a given monkey across time, (Farshchian *et al* 2019, Degenhart *et al* 2020, Karpowicz *et al* 2022, Ma *et al* 2023) but they have not yet been successfully applied across monkeys. These methods should be further investigated as a means to allow transfer decoding to be applied to a broader range of unstructured tasks.

In addition, because it separates the decoder computation and alignment phases, transfer decoding could take advantage of large quantities of more readily available data from monkeys, in principle, even across multiple monkeys. There is indeed evidence that a single decoder trained on a large dataset spanning many months helps the robustness of a BCI decoder over time (Sussillo *et al* 2016). With more extensive data, it may be possible to train even more sophisticated decoders that can operate across various behaviors, while relying on the more limited data from the human user for neural alignment. It is worth noting that, in this setting, the alignment step in the transfer approach might require less data from the target subject than direct decoding (figure S6). Although their effectiveness needs to be validated in future studies, deep learning-based decoders could be used for this purpose, as they have been shown to be more robust to variability in recording conditions and have higher decoding accuracy compared to linear alternatives (Glaser *et al* 2020, Willett *et al* 2021, 2023, Willsey *et al* 2022, Ye *et al* 2023). Using data from multiple source subjects could potentially improve performance for the direct decoding approach as well, though it would require good alignment between the behavior of all the sources and the target subject. However, as the range of motor actions becomes broader and the number of sources more numerous, this requirement becomes less likely.

## 5. Summary

To our knowledge, this study is the first that demonstrates the feasibility of cross-user EMG decoding, using both a ‘direct’ method and one that relies on the ‘transfer’ of a decoder computed from a monkey to a human user. We validated both approaches on multiple pairs of monkeys, and then tested them between a human with paralysis and a monkey. Future studies should focus on developing more sophisticated decoders and neural aligners to further expand the applicability of these approaches to varied and unstructured movements typical of daily living. While their feasibility would have to be tested online to address the problems associated with motor adaptation, the current study serves as an initial and encouraging proof of concept.

## Data Availability

The datasets for monkey J and S analyzed for this manuscript are publicly available at https://doi.org/10.5061/dryad.cvdncjt7n. The data from the human participant are available upon reasonable request from the authors.
